# Influence of NiTi alloy on the root canal shaping capabilities of the ProTaper Universal and ProTaper Gold rotary instrument systems

**DOI:** 10.1590/1678-77572016-0230

**Published:** 2017

**Authors:** Jussaro Alves DUQUE, Rodrigo Ricci VIVAN, Bruno Cavalini CAVENAGO, Pablo Andrés AMOROSO-SILVA, Ricardo Affonso BERNARDES, Bruno Carvalho de VASCONCELOS, Marco Antonio Hungaro DUARTE

**Affiliations:** 1 Universidade de São Paulo, Faculdade de Odontologia de Bauru, Departamento de Dentística, Endodontia e Materiais Odontológicos, Bauru, SP, Brasil.; 2 Universidade Federal do Paraná, Departamento de Odontologia, Curitiba, PR, Brasil.; 3 Associação Brasileira de Odontologia, Departamento de Endodontia, Taguatinga, DF, Brasil.; 4 Universidade Federal do Ceará, Departamento de Odontologia, Fortaleza, CE, Brasil.

**Keywords:** Endodontics, Micro-computed tomography, Nickel, Titanium, Root canal preparation

## Abstract

**Objective:**

This study aimed to evaluate the influence of the NiTi wire in Conventional NiTi (ProTaper Universal PTU) and Controlled Memory NiTi (ProTaper Gold PTG) instrument systems on the quality of root canal preparation.

**Material and Methods:**

Twelve mandibular molars with separate mesial canals were scanned using a high-definition microcomputed tomography system. The PTU and PTG instruments were used to shape twelve mesial canals each. The canals were scanned after preparation with F2 and F3 instruments of the PTU and PTG systems. The analyzed parameters included the remaining dentin thickness at the apical and cervical levels, root canal volume and untouched canal walls. Data was analyzed for statistical significance by the Friedman and Dunn’s tests. For the comparison of data between groups, the Mann-Whitney test was used.

**Results:**

In the pre-operative analysis, there were no statistically significant differences between the groups in terms of the area and volume of root canals (P>.05). There was also no statistically significant difference between the systems with respect to root canal volume after use of the F2 and F3 instruments. There was no statistical difference in the dentin thickness at the first apical level between, before and after instrumentation for both systems. At the 3 cervical levels, the PTG maintained centralization of the preparation on the transition between the F2 and F3 instruments, which did not occur with the PTU. Conclusion The Conventional NiTi (PTU) and Controlled Memory NiTi (PTG) instruments displayed comparable capabilities for shaping the straight mesial root canals of mandibular molars, although the PTG was better than the PTU at maintaining the centralization of the shape in the cervical portion.

## Introduction

Mechanical preparation of the root canal is an important step in endodontic treatment. The aim of this step is to remove vital or necrotic pulp tissue while simultaneously increasing the root canal volume to facilitate the decontamination of the root canal system by irrigants and medicaments^14^.

Several types of instruments and techniques for root canal preparations have been described^6,11,16-18^. Because of the known limitations of hand-held, stainless steel instruments (such as the risk of deviations in the root canal preparation and excessive time consumption), nickel-titanium (NiTi) instruments have been widely used in endodontic practice due to their relatively greater accuracy and efficiency compared to hand-held instruments^24^. NiTi rotary files have significantly better flexibility and cutting abilities and provide higher quality in the preparation of root canals with low risk of deviation^5^and higher fracture resistance to cyclic and torsional fatigue^25^.

NiTi wires have undergone various modifications in fabrication, including different types of thermic treatments that have been employed during the manufacturing process, which are designed to optimize physical characteristics that favor greater elasticity as well as increased flexibility and resistance^20^. Among the new NiTi wires now available on the market with the above-mentioned modifications, the CM wire is highlighted in the present study.

According to the manufacturers, CM wire instruments have a lower percentage of Nickel (by weight) than other NiTi instruments^10,22^. Additionally, because of the thermic treatments used during the manufacturing process, CM wire instruments do not bounce (rebound) to their original shape after being flexed. This characteristic, which is associated with greater flexibility, can improve the quality of the root canal preparation by reducing the risk of deviation, instrument fracture, and perforations^15^.

Among the NiTi systems, the PTU (Dentsply Maillefer, Ballaigues, Switzerland) is a rotary system of conventional NiTi wire that has been widely used and studied^1,6,11,24^. It has a variable taper along the length of the instrument, a convex triangular cross-section, and a sharp tip^7,13^. Another system, the PTG (Dentsply Maillefer, Ballaigues, Switzerland), was recently introduced to the market and has characteristics that are generally similar to those of the PTU, except that the PTG uses CM wire, while the PTU uses conventional NiTi wire. Because of this distinction, the PTG exhibits higher flexibility and greater resistance to cyclic fatigue than the PTU system^11,13^.

The aim of the present study was to evaluate the influence of the NiTi wire in PTU and PTG instruments on the quality of root canal preparation, as assessed by microcomputed tomography (micro CT). The null hypothesis evaluated was that the type of NiTi wire has no influence on the root canal preparation.

## Material and methods

### Selection of the teeth

One hundred extracted human mandibular molars with complete apexes were selected. The teeth were scanned using a high-definition micro CT system (Skyscan 1174v2, Bruker-microCT, Kontich, Belgium). Twelve teeth were selected based on the following attributes: root canal anatomy classified as Vertucci type IV^23^, with distinct main foramens, and maximum curvature of 5°^19^.

### Micro CT scanning procedures

The teeth were scanned as follows: 19 µm voxel size, 50 kV, 800 µA, and 0.8° step-size rotation using a 1024x1304 resolution. The obtained images were reconstructed with dedicated software (NRecon v.1.6.3; Bruker-microCT) and saved in axial sections in BMP format. The images were used to observe the root canal configuration and its initial volume.

### Root canal preparation

A single operator performed all procedures. The access cavities were conventionally performed, and the root canal lengths were determined by the insertion of #10 K-files (Dentsply Maillefer, Ballaigues, Switzerland) until visualization in the apical foramen with a stereomicroscope (Carl Zeiss Micro-imaging, Göttingen, Germany). The working length (WL) was established as being 1 mm shorter than the real root canal length (RL). The #10 and #15 K-Files (Dentsply Maillefer, Ballaigues, Switzerland) were used in the WL before instrumentation with the rotary systems. Next, 6 mesio-buccal and 6 mesio-lingual canals in different teeth (n=12) were instrumented using the PTU system as follows: S1 (18/.02) and SX (19/.035) instruments in the cervical preparation and the S1 (18/.02), S2 (20/.04), F1 (20/.07), and F2 (25/.08) instruments were used in the WL, according to the manufacturer’s instructions.

The remaining root canals (n=12) were instrumented using the PTG system in the same sequence as the one used for the PTU group. Subsequently, the roots were scanned again as in the initial evaluation. After scanning, the root canals were prepared with the F3 (30/.09) instrument of each system. The X-Smart Plus motor (Dentsply Maillefer, Ballaigues, Switzerland) was used with a specific torque and velocity for each instrument, in accordance with the manufacturer’s instructions, and the instrumentation was performed with in-and-out motion strokes in an apical direction. For the irrigation procedure, a 2.5% sodium hypochlorite solution was used; the root canals were irrigated before, during, and after instrumentation. Two milliliters of solution were used between each instrument change. The final irrigation was made with 3 mL of 17% EDTA for 3 minutes, and then with 3 mL of saline solution. Next, a new scan of the roots was performed using previously set parameters.

### Micro-CT measurements and evaluations

The CTAn v.1.12 software (Bruker-microCT) was used to measure the canal volumes as well as the dentin thickness in the mesial and distal walls in the cervical and apical portions. For pre- and post-operative measurements, images of 3 different slices of the cervical third were evaluated; the first slice was 1 mm below the level of bifurcation, and the other 2 slices followed apically at intervals of 1 mm. In the apical third, the first slice was obtained at 1 mm from the apical foramen and the other 2 slices followed cervically at 1 mm intervals. The minimal dentin thickness of the mesial and distal walls of the root canals was measured in millimeters. The CTAn v.1.12 software was used to measure the canal volume between the 1 mm apical level and at 1mm below the root furcation.

For analysis of the un-instrumented area, 3D models were created with color codes, and the pre- and post-operative images were recorded using automatic image registration. Prepared areas and stripes of the canals were compared using the CTVol v. 2.2.1 software (Bruker-microCT). The intact surface area of the canal was determined based on the number of static objects; i.e., markings present in the same position on the surface of the root canal before and after instrumentation^11^. The data was then converted and presented as: (a) percentage (%) of dentinal removal, (b) un-instrumented area, and (c) relative volume increase between groups.

The Shapiro-Wilks test was used to assess normality of the non-parametric data. Results of the volume increase, dentin thickness, and un-instrumented areas were compared using the Friedmann and Dunn’s tests for the intra-group analyses. For analysis of the percentage of dentinal wear, un-instrumented areas, and volume increase between the groups, the Mann-Whitney test was used. The level of significance was established at 5% (*P*<0.05).

## Results

**Figure 1:**
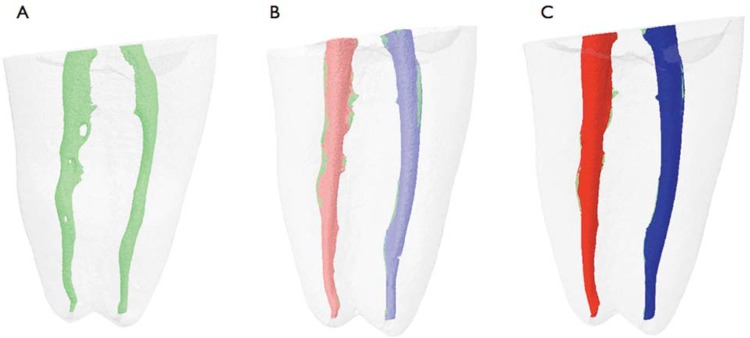
Three-dimensional reconstructions of mesial root canals of a mandibular molar: (A) before instrumentation (green), (B) after instrumentation with the ProTaper Gold F2 (light red), and the ProTaper Universal F2 file (light blue). (C) Shows the mesial canals after instrumentation with ProTaper Gold F3 (dark red) and the ProTaper Universal F3 file (dark blue)

In the pre-operative analysis, there were no statistically significant differences in the area or volume of root canals between the PTU and PTG groups (*P*>0.05), indicating adequate pairing of the root canals. Table 1 shows the median, minimum, and maximum values of the root canal volume (mm^3^) and surface area (mm^2^) before and after use of the F2 and F3 instruments. Also presented in Table 1 are the percentage of volume increase and the percentage of static voxel after use of the F2 and F3 instruments. No statistically significant differences were observed between the PTU and PTG systems with respect to the root canal volumes obtained after use of the F2 and F3 instruments (*P*>0.05). However, upon intra-group analysis of the PTU group, a statistically significant difference was observed between the pre-operative and post-operative volumes, but only after use of the F3 instrument (*P*<0.05). For the PTG group, a statistically significant difference was found between the volumes before and after use of the F2 and F3 instruments (*P*<0.05). Figure 1 depicts the three-dimensional reconstructions of mesial root canals before instrumentation (Figure 1A), and after instrumentation with F2 (Figure 1B) and F3 (Figure 1C) instruments.

**Table 1:** Median, minimum and maximum of the volume values (mm3), percentage of volume increase, surface area (mm2) and percentage of static voxel

	Protaper Universal			Protaper Gold		
Parameters	Initial	Post F2	Post F3	Initial	Post F2	Post F3
Volume mm^3^	1.094^Aa^ (0.511-2.164)	3.570^Aab^ (2.158-4.177)	3.915^Ab^ (2.319-4.477)	1.224^Aa^ (0.482-2.825)	3.640^Ab^ (1.874-4.440)	4.124^Ab^ (2.142-4.723)
% increase	-	64.73^Aa^ (36.74-86.95)	66.94^Ab^ (42.58-87.99)	-	60.39^Aa^ (31.65-86.41)	62.10^Ab^ (34.67-88.31)
Surface Area mm^2^	15.22^Aa^ (10.15-21.82)	24.17^Ab^ (17.36-28.26)	25.54^Ab^ (18.35-28.89)	17.70^Aa^ (11.66-28.12)	25.87^Aab^ (15.90-30.41)	27.76^Ab^ (16.76-30.57)
% static voxel	-	6.409^Aa^ (1.094-17.47)	4.813^Ab^ (0.118-11.250)	-	9.874^Aa^ (1.713-23.33)	6.152^Ab^ (1.224-17.74)

Different uppercase letters correspond to different values with statistical differences between groups and the same instrument (P<.05)

Different lowercase letters correspond to values with intra-group statistical differences (P<.05)

Table 2 shows the median, minimum and maximum values (presented as percentages) of dentin wear in the mesial and distal walls (mm) at the 3 apical and 3 cervical levels, before and after use of the F2 and F3 instruments for the PTU and PTG groups, respectively. There was no statistical difference between the percentage of dentin wear at the first apical level before and after instrumentation for both systems (*P*>0.05). At the 3 cervical levels, the PTG system maintained centralization of the preparation on transition between the F2 and F3 instruments, in contrast to the PTU system where this did not occur.

**Table 2:** Median, minimum and maximum percentage of mesial and distal wall wear at 3 apical and 3 cervical levels proportioned by the ProTaper Universal and ProTaper Gold instruments

ProTaper Gold						Protaper Gold					
	Level	Dentin wall	Post F2	Post F3	P mesial x distal		Level	Dentin wall	Post F2	Post F3	P mesial x distal
Apical	1	Mesial	4.129 (0-15.020)	0.930 (0-9.332)	Ini/F2 NS	Apical	1	Mesial	6.648 (2.172-12.830)	2.043 (0-8.989)	Ini/F2 NS
Apical		Distal	5.481 (0.838-20.570)	2.515 (0-11.090)	F2/F3 NS	Apical		Distal	3.448 (0-10.490)	0 (0-12.940)	F2/F3 NS
Apical	2	Mesial	8.011 (2.558-27.960)	1.754 (0-12.490)	Ini/F2 NS	Apical	2	Mesial	7.576 (2.243-20.980)	4.441 (0-16.640)	Ini/F2 p<.05
Apical		Distal	6.106 (2.775-23.770)	2.563 (0-14.800)	F2/F3 NS	Apical		Distal	3.729 (2.727-10.450)	2.107 (0-9.365)	F2/F3 p<.05
Apical	3	Mesial	8.662 (3.092-24.960)	3.614 (0-13.880)	Ini/F2 NS	Apical	3	Mesial	8.518 (3.980-19.980)	3.478 (0-12.580)	Ini/F2 NS
Apical		Distal	6.751 (2.049-22.690)	3.373 (0-8.549)	F2/F3 NS	Apical		Distal	5.948 (0-13.320)	3.348 (0-9.365)	F2/F3 NS
Cervical	1	Mesial	6.259 (0-14.490)	0.518 (0-5.286)	Ini/F2 p<.05	Cervical	1	Mesial	3.673 (1.816-21.950)	1.927 (0-6.041)	Ini/F2 p<.05
Cervical		Distal	31.890 (10.440-42.950)	4.728 (2.035-17.010)	F2/F3 p<.05	Cervical		Distal	25.920 (8.824-51.870)	3.211 (0-15.930)	F2/F3 NS
Cervical	2	Mesial	10.550 (0-15.770)	0.297 (0-6.431)*	Ini/F2 p<.05	Cervical	2	Mesial	5.369 (1.852-13.890)	2.332 (0-4.086)*	Ini/F2 p<.05
Cervical		Distal	37.100 (7.697-52.450)	6.186 (1.031-10.510)	F2/F3 p<.05	Cervical		Distal	30.840 (10.370-63.450)	4.855 (0-12.680)	F2/F3 NS
Cervical	3	Mesial	10.500 (1.289-20.710)	1.324 (0-6.693)	Ini/F2 p<.05	Cervical	3	Mesial	5.878 (1.149-25.240)	2.327 (0-5.891)	Ini/F2 p<.05
Cervical		Distal	24.070 (5.411-42.460)	6.452 (3.699-17.360)	F2/F3 p<.05	Cervical		Distal	22.280 (12.810-53.480)	5.648 (0-14.970)	F2/F3 NS

* statistically significant difference (p<.05)

**Figure 2:**
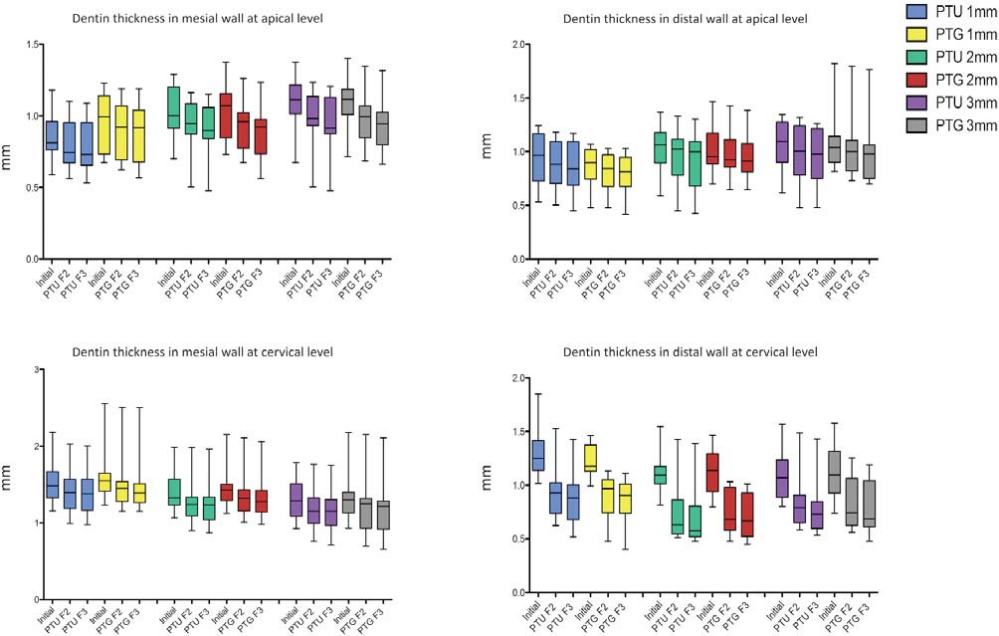
Graphic representation of the median, minimum and maximum values of the initial dentin thickness at the apical and cervical portions after use of the F2 and F3 instruments

For all segments that were evaluated, a dentin thickness of less than 0.4 mm was found regardless of the instrument and system used (Figure 2).

## Discussion

With respect to the specific root canal anatomy that was targeted for analysis in the present study, the data supports the null hypothesis because there were no statistically significant differences between the PTU and PTG instruments with respect to root canal enlargement or un-instrumented areas.

After a micro-CT evaluation, the mandibular molars to be examined were selected based on whether they: (a) had two canals in the mesial roots, (b) presented distinct foramens and (c) had no isthmuses related to type IV of Vertucci’s classification^23^. This experimental model allowed us to compare the capabilities of two instrument systems based on the same anatomical condition^18^.

Several methodologies such as histologic sections, serial axial sections, and scanning electron microscopy have been used to evaluate root canal preparations^2,4,8^; however, these methods compromise the integrity of the samples because they require the cutting of the specimens, which limits specimen use to a single analysis. By contrast, micro-CT is a nondestructive method that allows for the progressive evaluation and observation of the preparation instruments with different kinetics^18^. The evaluations conducted in the present study were made after the use of the F2 and F3 instruments.

Although rotary NiTi instruments are flexible, they can suffer flexural and/or torsional fatigue^13,14^. With technological advancement, however, new generations of instruments have been developed with different cross sections, diameters, tapers, and blades that have various cutting angles. Interestingly, the most recent advances have been directed towards improvements of the NiTi wire, whereby subtle changes in the proportion of metals, and in thermomechanical treatments have enabled the development of instruments with greater flexibility and fracture resistance when compared to their previous generations^4,10,13^. For instance, the CM wire is widely used in rotary instruments because it has greater flexibility than conventional NiTi wires^10,15,21,22^. The CM wire is manufactured using a complex heat treatment, with variations in wire composition; i.e., it has a lower percentage (52% by weight) of nickel than the most commonly used wire, which is composed of 54.5 to 57% of nickel (by weight)^21^. The PTU and PTG systems share a similar design, but they vary in the composition of the NiTi wire. One study assessed the shaping ability of these two systems, and found that the PTG produced less transportation and maintained more dentin than the PTU^11^.

Apical preparations with larger diameters have been suggested based on studies that showed that this approach facilitates more efficient irrigation in the apical region^3^, better infection control and improved quality of the root canal filling^9^. A previous report showed that, in the mesial roots of mandibular molars, the increase of apical preparation before use of the 0.40 mm instrument significantly increased the root canal volume without significantly reducing the dentin thickness in the danger zone^18^. In the present study, the apical preparation was performed until use of the F3 (30.09) instrument, which promoted a significant increase in the root canal volume. In both groups the preparation was centralized at the last apical millimeter; however, in the 3 cervical levels evaluated, the PTG system maintained centralized shaping on transition between the F2 and F3 instruments, which did not occur with the PTU. Nonetheless, both systems maintained significant degrees of mesial and distal dentin wall thicknesses.

Another study by Gagliardi, et al.^11^(2015) showed that the PTG was associated with less deviation than the PTU, which could be explained by the curvature of the specimens, as that study used moderately curved (25°–35°) mesial canals of mandibular molars, in contrast to the present study that used straight root canals. Therefore, the degree of curvature of the root canals appears to be a key determinant of the performance of the NiTi CM wire.

For both groups, un-instrumented areas of the root canals were observed in all specimens, demonstrating that the PTU and the PTG instruments are not as capable of performing a complete mechanical cleaning of the dentin walls as other NiTi rotary instruments^11,12,26^. The post-preparation percentage of un-instrumented areas prior to use of the F2 instrument was comparable to that obtained in a previous study that employed similar instruments and methodologies^11^; however, in the present study, the root canals were enlarged before use of the F3 instrument, which promoted a significant reduction in un-instrumented areas. This increase in diameter after preparation may be associated with better mechanical cleaning. The dentin thickness at the cervical levels was significantly reduced with the use of the F3 instrument in the PTU group, and the distal wall thickness appeared to be the most at risk. However, after use of the F3 instrument, the dentin thickness at the distal wall, in the cervical portion, was, on average, larger than 0.4 mm in both groups.

## Conclusions

The ProTaper Universal and ProTaper Gold systems exhibited similar capabilities for shaping the straight mesial root canals of mandibular molars. In the specific anatomical condition examined here, the final preparation with the F3 instrument, regardless of the system, enhanced the root canal volume and reduced the un-instrumented area. In conclusion, theControlled Memory NiTi ProTaper Gold PTG system was better at maintaining the centralization of shape in the cervical portion of the straight mesial root canals of mandibular molars than the conventional NiTi ProTaper Universal PTU system.
